# Systematic characterization of non‐coding RNAs in triple‐negative breast cancer

**DOI:** 10.1111/cpr.12801

**Published:** 2020-04-06

**Authors:** Jie Mei, Leiyu Hao, Huiyu Wang, Rui Xu, Yan Liu, Yichao Zhu, Chaoying Liu

**Affiliations:** ^1^ Department of Oncology Wuxi People's Hospital Affiliated to Nanjing Medical University Wuxi China; ^2^ Department of Physiology Nanjing Medical University Nanjing China; ^3^ State Key Laboratory of Reproductive Medicine Nanjing Medical University Nanjing China

## Abstract

Triple‐negative breast cancer (TNBC) is one of the most aggressive subtypes of breast cancer with negativity for oestrogen receptor (ER), progesterone receptor (PR) and human epidermal growth factor receptor (HER2). Non‐coding RNAs (ncRNAs) make up most of the transcriptome and are widely present in eukaryotic cells. In recent years, emerging evidence suggests that ncRNAs, mainly microRNAs (miRNAs), long ncRNAs (lncRNAs) and circular RNAs (circRNAs), play prominent roles in the tumorigenesis and development of TNBC, but the functions of most ncRNAs have not been fully described. In this review, we systematically elucidate the general characteristics and biogenesis of miRNAs, lncRNAs and circRNAs, discuss the emerging functions of these ncRNAs in TNBC and present future perspectives in clinical practice.

## 1 INTRODUCTION

In the past few decades, the morbidity of human breast cancer has increased continuously and has led to a great threat to women's lives. According to the statistics gathered by the American Cancer Society, there will be more than 271 000 new cases of breast cancer and approximately 42 260 deaths in 2019.^1^ Being a heterogeneous disease, breast cancer can be classified into several main subclasses based on the expression status of oestrogen receptor (ER), progesterone receptor (PR), human epidermal growth factor receptor (HER2) and antigen ki‐67 (Ki‐67).^2^ Among known subclasses, triple‐negative breast cancer (TNBC) is the most aggressive subtype, which is characterized by negativity for ER, PR and HER2. Great efforts have been made to understand the mechanisms of TNBC carcinogenesis, especially focus on the role of non‐coding RNAs (ncRNAs).

Non‐coding RNAs make up most of the transcriptome, while protein‐coding RNAs only account for 3% of the genome; the remaining 97% is composed of “dark matter” of transcripts with molecular functions.^3^ It has been proven that the genome “dark matter” can be transcribed into various RNA species, most of which do not encode proteins, namely, ncRNAs, but exert significant functions mainly responsible for phenotypic regulation.^4^ The emerging functions of ncRNAs have been generally determined in the cancer research field. Currently, studies of ncRNA‐related cancer are commonly concentrated on miRNAs, lncRNAs and circRNAs. Here, we review the general characteristics and functions of ncRNAs and discuss their underlying mechanisms in the carcinogenesis and development of TNBC.

## 2 CATEGORIES AND GENERAL CHARACTERISTICS OF NCRNAs

Depending on the number of nucleotides (nt), ncRNAs can be categorized into two main groups: (a) short ncRNAs, which include microRNA (miRNA), small interfering RNA (siRNA), small nucleolar RNA (snoRNA), small nuclear RNA (snRNA), piwi‐interacting RNA (piRNA), tRNA‐derived stress‐induced RNA (tiRNA) and tRNA‐derived small non‐coding RNA (tDR); (b) long ncRNAs (lncRNAs), which have transcripts with more than 200 nt in length and include long intergenic non‐coding RNA (lincRNA), natural antisense transcript (NAT), circular RNA (circRNA), pseudogene transcript, transcribed ultraconserved region (T‐UCR) and telomerase RNA component (TERC).^3^ Although circRNA belonging to the lncRNA family, researchers tend to discuss them separately distinguishing from lncRNAs due to their unique structure. The general characteristics and functions of common ncRNAs are summarized in Table 1.

**Table 1:** The main types of non‐coding RNAs and their features

Types of ncRNAs	Abbreviation	Length (nt)	Localization	Main functions
Short ncRNAs				
MicroRNA	miRNA	20‐24	Nucleus, cytoplasm	Translation suppression
Small interfering RNA	siRNA	20‐30	Cytoplasm	Translation suppression
Small nucleolar RNA	snoRNA	60‐200	Nucleus, cytoplasm	2’‐O‐methylation and pseudouridylation of rRNA
Small nuclear/cytoplasmic RNA	snRNA/scRNA	100‐200	Nucleus, cytoplasm	Component of spliceosome
piwi‐interacting RNA	piRNA	24‐31	Cytoplasm	Translation suppression, modulation of transposons
tRNA‐derived stress‐induced RNA	tiRNA	30‐40	Cytoplasm	Translation suppression, signalling molecule
tRNA‐derived small non‐coding RNA	TDRs	20	nucleus	Translation suppression, target transportable element
Long ncRNAs				
Long intergenic non‐coding RNA	lincRNA	>200	Nucleus, cytoplasm	miRNA sponge, regulation of gene transcription
Natural antisense transcript	NAT	>200	Nucleus, cytoplasm	inhibition of the mRNA, epigenetic gene silencing
Circular RNA	circRNA	200‐800	Nucleus, cytoplasm	miRNA sponge, regulation of gene transcription
Pseudogene transcript	—	>200	Nucleus, cytoplasm	translation repression, miRNA sponge
Transcribed ultraconserved region	T‐UCR	>200	Cytoplasm	miRNA sponge
Telomerase RNA component	TERC	451	Nucleus	Telomere length maintenance

## 3 MICRORNAs

### 3.1 Biogenesis of miRNAs

**Figure 1:**
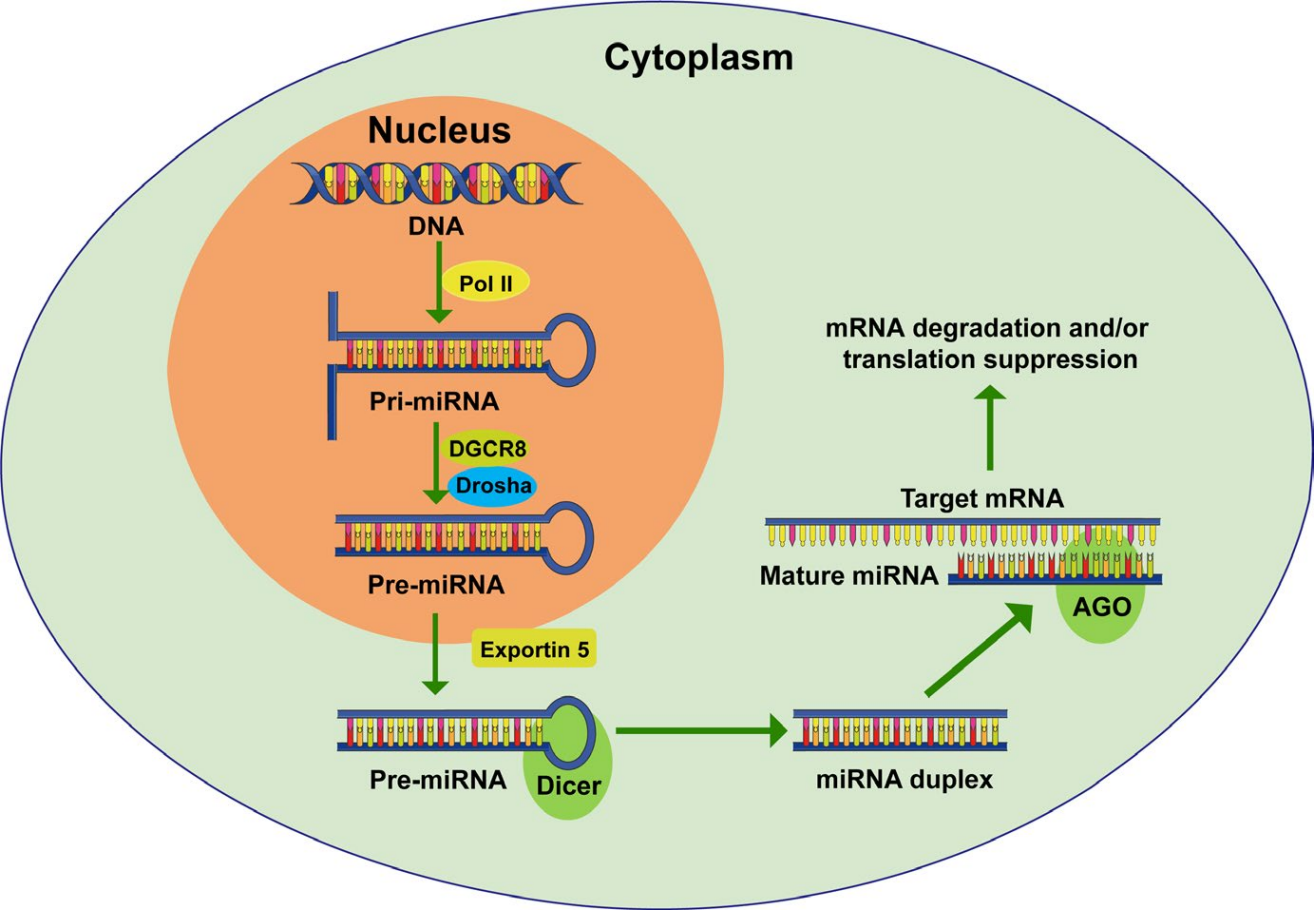
Mechanism of miRNA biogenesis. The miRNA gene is preliminarily transcribed into primary miRNA (pri‐miRNA, 300‐1000 bp) in the nucleus. A processing complex consisting of Drosha and DGCR8 cleaves the pri‐miRNA, resulting in the generation of precursor miRNA (pre‐miRNA, 70 bp). Exportin 5 exports the pre‐miRNA from the nucleus to the cytoplasm. In the cytoplasm, the pre‐miRNA is further processed by the Dicer complex into double‐stranded miRNA. One strand of the miRNA duplex (mature miRNA, 20‐24 nt) is selected to assemble the RNA‐induced silencing complex (RISC). The miRISC includes the miRNA, corresponding mRNA and Argonaute (AGO) and causes mRNA destabilization and translational inhibition relying on miRNA binding to the complementary sequence in the 3'UTR of its target mRNA

MicroRNAs are endogenous, non‐coding small RNAs with approximately 20‐24 nt in length which mainly participate in regulating gene expression. In the canonical biogenesis pathway, genes encoding miRNAs are transcribed into long initial transcript by RNA polymerase Ⅱ, namely pri‐miRNAs, 300‐1000 base pairs (bp) in length.^5^ The pri‐miRNAs are cleaved to pre‐miRNAs (about 70 bp in length) with a stem‐loop structure under the action of RNase III Drosha.^6^ Next, the pre‐miRNAs are transported from the nucleus to the cytoplasm by the Ran‐GTP‐dependent transporter Exportin 5.^7^ The pre‐miRNAs are further processed by Dicer enzyme, a double‐stranded RNA‐specific RNA endonuclease, cleaving into 20‐24 nt double‐stranded miRNAs. To act as gene expression regulators, firstly, the mature miRNA binds to Dicer's complementary sequence to form a double helix, then the double helix unwinds, one of which with the lower stability in the 5' end preferentially binds to the RNA‐induced silencing complex (RISC) to form a complex including Argonaute (AGO) proteins, the mRNA strand and several cofactors. At last, the complex would combine its target mRNA, causing the inhibition of gene expression (Figure 1).

### 3.2 Functions of miRNAs

#### 3.2.1 MiRNAs cause mRNA degradation

MicroRNAs play significant regulatory roles in eukaryotes by binding to corresponding mRNA transcripts, leading to their degradation at mRNA level and/or translational repression. There is currently no clear conclusion on how miRNAs induce mRNA degradation. Two different perspectives have been issued currently: (a) ordinary mechanism of mRNA degradation. This view holds that miRNAs merely act as guides, they do not directly degrade mRNAs through AGO proteins, but only label and send mRNAs that need to be degraded to the normal mRNA degradation mechanism. Giraldez et al^8^ found that miR‐430 induced the deadenylation of its targets, resulting in the recruitment to processing bodies, where mRNAs were decapped and degraded. (b) MiRISC‐mediated mRNA degradation. This view holds that a variety of related enzymes and complexes, such as AGO proteins, decapping enzymes and their cofactors, all of which may be involved in miRNA‐mediated degradation of mRNAs. As a recognized mechanism, miRNAs bind to AGO proteins in miRISCs to recognize their mRNA targets. AGO proteins interact with a GW182 protein, in turn, interacts with cytoplasmic poly(A)‐binding protein (PABP) and with the cytoplasmic deadenylase complexes CCR4–NOT and PAN2‐PAN3, which catalyse the deadenylation of the mRNA targets, and then, deadenylated mRNAs are decapped and rapidly degraded by 5’ to 3’ exoribonuclease 1 (XRN1).^9^, ^10^, ^11^

#### 3.2.2 MiRNAs repress protein translation

Several studies have proven that miRNA‐mediated gene silencing is first manifested through repressing translation and subsequently consolidated by mRNA deadenylation and decay.^12^, ^13^ Interestingly, Beilharz et al^14^ observed that miRNA‐mediated mRNA deadenylation contributed to translational repression in mammalian cells as well. Multiple perspectives have been issued to explain this mechanism, such as inhibition of ribosome assembly and degradation of neopeptide,^15^ but there are still no unified views up to now. The emerging recognition is that miRNAs inhibited translation initiation by interfering with the activity and/or assembly of the eukaryotic initiation factor 4F (eIF4F) complex, consisting of the cap‐binding protein eIF4E, the adaptor protein eIF4G and the box protein 6 (DEAD) box RNA helicase eIF4A.^16^ Whatever, it is now widely accepted that both mRNA degradation and translational repression participate in miRNA‐mediated gene silencing.

#### 3.2.3 Unconventional functions of miRNAs

Although negative regulation of target gene expression is the most common mechanism of miRNAs participating in biological functions, other non‐canonical mechanisms also play irreplaceable roles. It has been proven that miRNAs can interact with non‐AGO proteins. Eiring et al^17^ found that miR‐328 interacted with hnRNP E2, leading to release of CEBPA mRNA from hnRNP E2‐mediated translational inhibition. To our surprise, miRNAs can directly activate transcription. Matsui et al^18^ found that miR‐589, in complex with AGO2 and GW182, bound the promoter RNA of COX2, leading to transcriptional activation of COX2. Besides, other non‐canonical mechanisms were observed as well, including upregulating protein expression, activating Toll‐like receptors, targeting mitochondrial transcripts and targeting nuclear ncRNAs.^19^

### 3.3 MiRNAs in TNBC

MicroRNAs have been exhibited to act as multiple roles in TNBC, including functioning as diagnostic and prognostic biomarkers and exerting multifaceted effects on tumour progression, such as the modulation of tumour growth, metastasis and manipulation of chemoresistance, and regulation of metabolism (Table 2).

**Table 2:** Summarization of the cellular functions of miRNAs in tumorigenesis of TNBC

MiRNAs	Role in TNBC	Cancer phenotype	Target gene	References
miR‐498	Oncogene	Promoted proliferation and migration	PTEN	^37^
miR‐374a‐5p	Oncogene	Promoted proliferation and migration	ARRB1	^38^
miR‐761	Oncogene	Promoted proliferation migration and invasion	TRIM29	^39^
miR‐106b‐25 cluster	Oncogene	Induced tumour initiating cell phenotypes	NEDD4L	^40^
miR‐199	Oncogene	Promoted proliferation and migration	/	^41^
miR‐214	Oncogene	Promoted proliferation and migration	/	^41^
miR‐200c	Oncogene	Promoted migration and invasion	/	^46^
miR‐423‐5p	Oncogene	Promoted migration and invasion, increased resistance to cisplatin	/	^58^
miR‐18a	Oncogene	increased resistance to cisplatin	Dicer	^60^
miR‐181a	Oncogene	Promoted migration and invasion, increased resistance to doxorubicin	BAX	^62^
miR‐221	Oncogene	Promoted proliferation, migration and invasion	PTEN	^66^
miR‐939	Oncogene	Increased tumour cell trans‐endothelial migration	VE‐cadherin	^67^
miR‐205	Tumour suppressor	Inhibited proliferation, cell cycle progression	E2F1, LAMC1	^33^
miR‐17‐5p	Tumour suppressor	Inhibited proliferation and migration	ETV1	^34^
miR‐3178	Tumour suppressor	Inhibited proliferation, invasion and migration	NOTCH1	^35^
miR‐185	Tumour suppressor	Inhibited proliferation	E2F6, DNMT1	^36^
miR‐200a	Tumour suppressor	Inhibited migration	EPHA2	^44^
miR‐200b	Tumour suppressor	Inhibited migration and invasion	PKCα, ARHGAP18	45, 55
miR‐655	Tumour suppressor	Inhibited migration and invasion	PRRX1	^48^
miR‐125b	Tumour suppressor	Inhibited proliferation, migration and invasion	MAP2K7	^49^
miR‐212‐5p	Tumour suppressor	Inhibited migration and invasion	PRRX2	^50^
miR‐613	Tumour suppressor	Inhibited migration and invasion	DAAM1	^51^
miR‐638	Tumour suppressor	Inhibited proliferation and migration, increased sensitivity to cisplatin	BRCA1	^57^
miR‐101	Tumour suppressor	Inhibited proliferation, induces apoptosis, increased sensitivity to paclitaxel	MCL1	^59^
miR‐130a‐3p	Tumour suppressor	Increased sensitivity to doxorubicin	/	^61^
miR‐451a	Tumour suppressor	Increased sensitivity to doxorubicin	/	^61^
miR‐34a	Tumour suppressor	Inhibited proliferation and invasion, increased sensitivity to dasatinib	c‐SRC	^64^

#### 3.3.1 MiRNAs function as diagnostic and prognostic biomarkers

Most functional miRNAs tend to exhibit various expression patterns between TNBC patients and healthy individuals even other subtype breast cancer samples. Furthermore, miRNAs can indicate cancer progression and monitor the prognosis of TNBC patients, which have potential to act as biomarkers in clinical practice.

Circulating cell‐free miRNAs extracted from blood are a hotspot in the field of diagnostic biomarkers.^20^ According to published literature, circulating cell‐free miR‐199a‐5p could function as a TNBC‐specific diagnostic biomarker distinguishing TNBC patients from non‐TNBC and healthy controls.^21^ Circulating miR‐105 and miR‐93‐3p also acted as a diagnostic biomarker for TNBC.^22^ Niedźwiecki et al^23^ found that the expression level of miR‐200c was lower in TNBC patients' serum when compared with the levels in the ER/PR‐positive group, revealing that miR‐200c is a diagnostic biomarker to distinguish the subtype of breast cancer.

Since the prognosis of TNBC patients is quite poor, prognostic evaluation is essential for both clinicians and patients. Positive miR‐34b, miR‐301a and miR‐361‐5p expression was significantly associated with poor overall survival (OS) in TNBC patients.^24^, ^25^, ^26^ In addition, high expression of miR‐9 and miR‐155 exhibited a tight association with poor distant metastasis‐free survival (DMFS) in TNBC patients, and the level of miR‐9 expression also correlated with worse disease‐free survival (DFS).^27^ On the other hand, several miRNAs have been shown to function as tumour suppressors in TNBC. MiR‐148a suppressed metastasis of TNBC and served as a prognostic indicator.^28^ Moreover, stroma‐derived miRNAs exhibited promising value in the prognostic evaluation of TNBC. Stromal expression of miR‐21 was remarkably upregulated and associated with recurrence‐free survival (RFS).^29^

Furthermore, prognostic models developed based on combinations of multiple miRNA signatures have also been shown excellent values. Two independent miRNA signatures developed by Cascione et al^30^ had predictive values for OS and DMFS in TNBC patients, respectively. In addition, a prognostic model developed based on the expression levels of miR‐155, miR‐493, miR‐30e and miR‐27a accurately identified high‐risk and low‐risk groups in TNBC patients’ cohort.^31^ Besides, Kleivi et al^32^ performed genome‐wide serum miRNA expression detection and identified a four‐miRNA signature that predicted RFS and OS; this prediction model may bring better treatment options for patients with TNBC.

#### 3.3.2 MiRNAs modulate cancer cell growth

Cell proliferation is crucial for cancer progression and is commonly mediated by the dysregulation of cell cycle–related proteins. Tumour growth tends to contribute to the occurrence of tumour angiogenesis and metastasis. Emerging studies have shown that miRNAs regulate cell proliferation in TNBC.

Loss of p53, a classic tumour suppressor, always occurs in tumours. MiR‐205 is directly transactivated by p53 and is downregulated in TNBC. Re‐expression of miR‐205 significantly reduced cell proliferation in vitro and inhibited tumour growth in vivo. The underlying mechanism by which miR‐205 inhibited cancer cell growth was that overexpressed miR‐205 directly targeted E2F1 to regulate cell cycle progression and LAMC1, thus modulating cell adhesion, proliferation and migration.^33^ More targets regulated by miRNAs in TNBC have recently been revealed. Re‐expression of the silenced miRNAs miR‐17‐5p and miR‐3178 in TNBC inhibited cell proliferation and migration by targeting ETV1 and NOTCH1, respectively.^34^, ^35^ In addition, miR‐185 suppressed tumour proliferation by directly downregulating E2F6 and DNMT1, thus indirectly upregulating BRCA1 in TNBC.^36^

On the other hand, oncogenic miRNAs also play crucial roles in TNBC. MiR‐498, miR‐374a‐5p and miR‐761 were significantly overexpressed in TNBC and promoted cell proliferation and migration by downregulating PTEN, ARRB1 and TRIM29, respectively.^37^, ^38^, ^39^ Moreover, the miR‐106b‐25 cluster mediated oncogenesis in breast cancer by activation of NOTCH1 by targeting NEDD4L in both ER+ and TNBC breast cancer cells, suggesting that the miR‐106b‐25/NEDD4L/NOTCH1 axis played a crucial role in breast cancer.^40^ Cantini et al described a novel algorithm named ClustMMRA, which was applied to explore clustered miRNAs potentially driving cancer molecular subtypes. Using ClustMMRA to analyse breast cancer patient data, the authors identified miR‐199/miR‐214 as a novel cluster that promoted TNBC cell proliferation and further validated these findings in vitro.^41^

#### 3.3.3 MiRNAs affect migration and metastasis

Although the aetiology and onco‐genetic mechanism of TNBC have been initially investigated, there are no defined conclusions to explain metastasis in clinical practice, which causes thousands of cancer‐related deaths. Emerging evidence has demonstrated that miRNAs are associated with the metastatic process in TNBC, suggesting novel strategies to control metastasis and improve prognosis.

Emerging evidence suggests that miRNAs are associated with the metastatic process in TNBC. Dysregulated expression of miR‐200 family members has been observed in multiple cancers.^42^, ^43^ However, the roles of miR‐200 family members in the metastasis of breast cancer are still disputable. MiR‐200a and miR‐200b inhibited the migration of TNBC cells by directly targeting EPHA2 and PKCα, respectively.^44^, ^45^ In contrast, overexpression of miR‐200c promoted the migration and invasion capacity of TNBC cells by secreting VEGF‐A through activation of the FAK and PI3K/AKT signalling pathways.^46^ Epithelial‐mesenchymal transition (EMT) is a critical process in cancer cell invasion, characterized by the downregulation of cell adhesion markers with the concomitant acquisition of mesenchymal molecules.^47^ MiRNAs serve as crucial regulators in the EMT process, and miR‐655, miR‐125b and miR‐212‐5p have been reported to suppress the EMT process by targeting PRRX1, MAP2K7 and PRRX2 in TNBC, respectively.^48^, ^49^, ^50^

RhoA is a classic small GTPase that is generally thought to be essential for the formation of stress fibres and invasion of cancer cells. Xiong et al^51^ reported that miR‐613 suppressed TNBC cell migration and invasion by targeting DAAM1, a novel indirect regulator of RhoA activation.^52^, ^53^, ^54^ However, to our surprise, Humphries et al^55^ found that downregulation of ARHGAP18 by miR‐200b inhibited metastasis of TNBC by increasing the activation of RhoA, suggesting the tumour suppressive role of RhoA in TNBC.

#### 3.3.4 MiRNAs regulate the sensitivity to therapeutic drugs

Chemotherapy remains an indispensable therapeutic strategy for TNBC, but the acquisition of chemoresistance is the primary obstacle for successful treatment. Accordingly, tremendous effort has been made in recent years to elucidate the mechanisms of TNBC chemoresistance with the aim of identifying new molecular targets.^56^ It has become evident that ncRNAs act as significant regulators in the development of TNBC chemoresistance.

Cisplatin is a common anti‐cancer drug applied in clinical practice. Overexpression of miR‐638 increased sensitivity to cisplatin by regulating the expression of BRCA1 in TNBC.^57^ In contrast, miRNAs could also promote resistance to chemotherapy. Cisplatin‐resistant MDA‐MB‐231 cell‐derived miR‐423‐5p increased the resistance of recipient cells in an exosomal‐dependent manner.^58^ The bidirectional effects of miRNAs on chemoresistance have also been demonstrated for paclitaxel and doxorubicin. MiR‐101 increased paclitaxel sensitivity by downregulating MCL1 expression,^59^ while miR‐18a conferred paclitaxel resistance by targeting Dicer in TNBC.^60^ Furthermore, overexpression of miR‐130a‐3p and miR‐451a in TNBC cells significantly enhanced the cell sensitivity to doxorubicin,^61^ but high expression levels of miR‐181a contributed to chemotherapy resistance and predicted poor DFS and OS in patients receiving these treatments.^62^ MiRNAs also regulate the curative effect of targeted therapies. Decreased miR‐206 expression was observed in BRCA1 wild‐type TNBC cells after concomitant treatment with gemcitabine and PARP1 inhibitor, suggesting that miR‐206 served as a curative regulator of PARP1 inhibitor combination chemotherapy for BRCA1 wild‐type TNBC patients.^63^ Expression of miR‐34a was silenced in TNBC, and re‐expressed miR‐34a in cell lines promoted sensitivity to dasatinib and suppressed proliferation and invasion by downregulating c‐SRC.^64^

#### 3.3.5 Exosomes‐mediated miRNAs in TNBC

Exosomes are becoming a novel hotspot in the field of cancer research, which are a subclass of extracellular vesicles involved in intercellular communication.^65^ Not surprisingly, exosomes‐mediated miRNAs play significant roles in tumorigenesis of TNBC. As described before, cisplatin‐resistant MDA‐MB‐231 cells transmitted resistance by exosomes containing miR‐423‐5p.^58^ In addition, cancer cell‐derived exosomes have been shown to transfer miR‐221 to recipient cells to promote EMT, thereby promoting metastasis.^66^ Modica et al^67^ observed the extracellular pro‐tumorigenic role of tumour‐derived, exosome‐associated miR‐939 in targeting VE‐cadherin, explaining its association with poor prognosis in TNBC. Moreover, exosomal miRNAs could serve as monitoring indicators for therapeutic response. Stevic et al^68^ found that 17 miRNAs in 224 TNBC patients who underwent neoadjuvant therapy were significantly deregulated, which were significantly associated with the clinic‐pathological and risk factors. To sum up, as a novel hotspot of cancer research, exosome‐mediated miRNAs may act as multiple roles in TNBC, which should be further explored in the future.

## 4 LONG NON‐CODING RNAs

### 4.1 Biogenesis of lncRNAs

**Figure 2:**
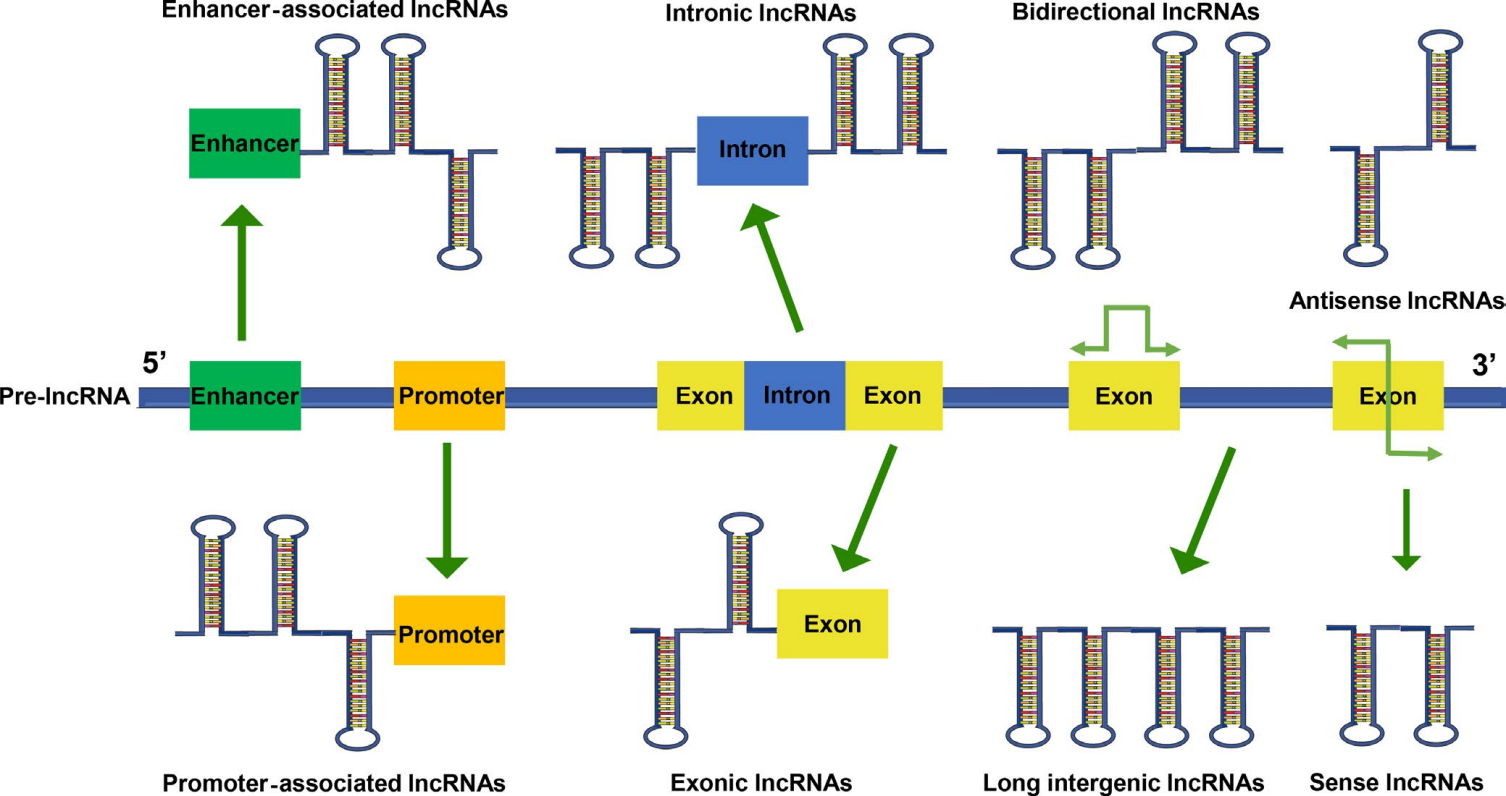
Mechanism of lncRNA biogenesis. LncRNA is transcribed by RNA polymerase II, capped at the 5' end, polyadenylated at the 3' end and edited by a series of splicing events that occur in the nucleus. This figure preliminarily summarizes the diverse biogenesis of lncRNAs based on their transcriptional origin, namely, promoter‐associated lncRNAs, enhancer‐associated lncRNAs, exonic lncRNAs, intronic lncRNAs, bidirectional lncRNAs, intergenic lncRNAs, antisense lncRNAs and sense lncRNAs

LncRNA is one of ncRNAs subclass that most researched in the field of cancer research. In mammalian genome, more than 58 000 lncRNAs have been identified, and approximately 30 000 lncRNAs have been curated in GENCODE v29.^69^ As we all know, lncRNAs exist widely in human organism, and it is vital to human gene expression modulation and physiological and pathological processes. Most types of lncRNAs are transcribed by RNA polymerase Ⅱ, capped at the 5' end, polyadenylated at the 3' end and edited by a series of splicing processes which occurs in the nucleus. Besides, there are other mechanisms participating in the process of lncRNA maturation. For instance, precursor lncRNAs (pre‐lncRNAs) are cleaved by RNaseP to achieve mature ends.^70^ LncRNAs are pervasively interspersed in the genome based on their various transcriptional origins, including whole or partial natural antisense transcripts, coding genes, between coding genes, within introns, promoter and enhancer (Figure 2). Due to the complexity and diversity of lncRNAs, the biogenesis and regulation mechanism of different lncRNAs have not been completely summarized. In the future years, by virtue of the advanced techniques, including chromatin isolation by ChIRP‐Seq, CRISPR and CLIP,^71^, ^72^, ^73^ it is worthy of believing that the mechanism of biogenesis and synthesis of lncRNAs will be further deeply cognized.

### 4.2 Functions of lncRNAs

#### 4.2.1 LncRNAs act as ceRNA

Long ncRNAs have diversity of regulatory functions, which can modulate chromatin remodelling, transcriptional regulation and post‐transcriptional processing, translation.^74^, ^75^ Although multiple functions of lncRNAs have been observed, competing endogenous RNA (ceRNA) or miRNA sponge is the most focused function. For instance, LINC01123 was found to be upregulated in non‐small‐cell lung cancer and associated with poor prognosis. By a series of functional experiment, researchers revealed that LINC01123 might increase c‐MYC mRNA expression by sponging miR‐199a‐5p, which was a direct transcriptional regulator for c‐MYC.^76^ Even if the function of lncRNAs as ceRNA has been widely accepted, the mechanism concerning these remain unknown to some extent and require to further deeper investigation.

#### 4.2.2 LncRNAs modulate transcriptional process

Long ncRNAs can directly participate in mediating transcription by different mechanisms.^77^ R‐loops are defined as a novel DNA‐RNA hybrid abundant at CpG islands, and it can be formed when the nascent RNA reanneals into the template DNA.^78^ Arab et al^79^ uncovered that lncRNA TARID could form an R‐loop at the TCF21 promoter and regulated its transcriptional level. Causally, there is a CG skew within exon 2 of TARID, rending TARID overlap with CpG islands around the transcription start site of TCF21. By DRIP‐qPCR assay, it revealed that R‐loops could coincide with the TSS‐proximal GC skew. When silencing TCF21 by promoter hypermethylation or losing the TARID promoter, the R‐loop structure was not observed in cells. In addition, upregulation of RNase H1 degrading RNA within DNA‐RNA hybrids would reduce the level of R‐loops at TCF21 and the level of TARID. Moreover, lncRNAs can regulate transcription through binding to histone‐modifying complexes, to transcription factors and even to RNA polymerase II as well.^80^

#### 4.2.3 LncRNAs modulate translation

Distinctly, lncRNAs are defined as a sort of ncRNA without translation function. However, during the process of translation, lncRNAs play indispensable roles. As recently reviewed, it has been found that lncRNA MEG3 significantly decreased in human invasive bladder cancers, and its exogenous expression can inhibit the invasiveness of human bladder cancer cells. The main reason is that MEG3 negatively regulated the expression of c‐MYC by promoting PHLPP2 protein translation.^81^ In recent years, ribosome profiling has identified ribosome‐associated lncRNAs, which accords with the idea that some annotated lncRNAs may be translated, which remains under investigation. Besides, some lncRNAs can be used as a scaffold for assembly of whole protein complexes, for example NEAT1 for paraspeckle proteins and HOTAIR for the HBXIP/HOTAIR/LSD1 complex.^82^, ^83^

#### 4.2.4 LncRNAs are involved in chromatin action

There are lots of lncRNAs accumulating in the nucleus to regulate chromatin architecture, participate in chromatin remodelling and promote the recruitment of chromatin modifiers. For example, the genome multipotent stem cells are equipped with the form of higher‐order chromatin architecture, with a variety of intra‐ and inter‐chromosomal interactions. The peculiarity of stem cell pluripotency is directly determined by the promoter region of stem cell core factor genes around the architecture. In many studies, lncRNAs can take part in the composition of chromatin architecture to establish the stemness state, chiefly bringing distant enhancer elements into proximity of the core promoter.^84^ Besides, lncRNAs also can interact with chromatin‐modifying enzymes, catalysing covalent changes of histones or DNA on the chromatin to affect genetic expression information, such as GADD45a, DNMT1 and PRC.^85^

#### 4.2.5 LncRNAs regulate mRNA stability

Studies show that lncRNAs can regulate mRNA stability via corresponding miRNAs, recruiting proteins to degrade mRNA and acting as molecular decoys for RBPs taking part in mRNA decay. For instance, the binding of hnRNP E1 to a nucleic acid structural element located in exon 12 of PNUTS pre‐RNA can regulate its alternative splicing. In breast cancer cells, the expression of lncRNA PNUTS was elevated and associated with levels of ZEB mRNAs. Furthermore, PNUTS also served as a competitive sponge for miR‐205 during epithelial‐mesenchymal transition.^86^ Besides, PDCD4‐AS1 regulated breast cancer progression through stabilizing PDCD4 RNA by forming RNA duplex and controlling the interaction between PDCD4 RNA and RNA decay‐promoting factors such as HuR.^87^ Although the role of lncRNAs in regulating mRNA stability has been rarely observed, it is a novel mechanism that lncRNA‐related biological process needs to be further explored in the future.

### 4.3 LncRNAs in TNBC

Long ncRNAs play crucial roles in human beings, not only do lncRNAs participate in regulating normal mammalian gene expression function or other body biological processes, but also they have notable influences on human diseases, such as various cancers, neuropsychiatric disorders and atherosclerosis.^88^, ^89^, ^90^ Apparently, there are gradually increasing explorations in the mechanism of lncRNAs functioning in TNBC in recent years. Accumulated evidence suggested that lncRNAs can modulate proliferation, invasion, differentiation, chemoresistance of TNBC cells in positive or negative manner (Table 3). Even a few studies indicated that lncRNAs can work as a biomarker for diagnosing and evaluating the prognosis in TNBC.

**Table 3:** Summarization of the cellular functions of lncRNAs in tumorigenesis of TNBC

LncRNAs	Role in TNBC	Cancer phenotype	Molecular mechanism	References
CCAT1	Oncogene	Promoted proliferation and migration	Regulated miR‐218/ZFX axis	^91^
TROJAN	Oncogene	Promoted proliferation	Induced ZMYND8 degradation	^92^
LINC00339	Oncogene	Promoted proliferation and inhibited apoptosis	Regulated miR‐377‐3p/HOXC6 axis	^93^
MIR100HG	Oncogene	Promoted proliferation and induced cell arrest in the G1 phase	Negatively regulated p27 gene expression	^94^
NRAD1	Oncogene	Promoted proliferation	Positively regulated by ALDH1A3	^95^
DANCR	Oncogene	Promoted proliferation	Bound to RXRA and enhanced PI3K/AKT signals	^96^
NAMPT‐AS	Oncogene	Promoted migration	Recruited POU2F2 to activate NAMPT Regulated miR‐548b‐3p/NAMPT axis	^10^
ROR	Oncogene	Promoted migration	Regulated miR‐145/ARF6 axis	^11^
Linc‐ZNF469‐3	Oncogene	Promoted migration	Regulated miR‐574‐5p/ZEB1 axis	^12^
HULC	Oncogene	Promoted migration	Upregulated MMP2 and MMP9	^13^
NEAT1	Oncogene	Inhibited apoptosis, regulated cell cycle progression and promoted chemoresistance	Upregulated ATP7A and ATP7B	^16^
BORG	Oncogene	Increased resistance to doxorubicin	Activated RPA1 and NF‐κB signals	^17^
HCP5	Oncogene	Increased resistance to cisplatin	Downregulated PTEN	^18^
SONE	Tumour suppressor	Inhibited proliferation and migration	Positively regulated TP53 and negatively regulated c‐MYC	^97^
RMST	Tumour suppressor	Inhibited proliferation and migration, induced apoptosis	/	^98^
PTCSC3	Tumour suppressor	Inhibited proliferation	Downregulated lncRNA H19	^99^
LOC554202	Tumour suppressor	Inhibited migration	Promoter hypermethylated	^14^
NEF	Tumour suppressor	Inhibited migration	Negatively regulated miR‐155	^15^
GAS5	Tumour suppressor	Increased sensitivity to paclitaxel, induced apoptosis	Regulated miR‐378a‐5p/SUFU axis	^19^

#### 4.3.1 LncRNAs regulate cancer cell growth

Long ncRNA CCAT1 expression is higher in TNBC cells than in normal breast epithelial cells. Functional analysis indicated that silencing CCAT1 inhibited cell proliferation and migration in TNBC by regulating the miR‐218/ZFX axis.^91^ Similarly, lncRNA TROJAN promoted the proliferation of TNBC cells by inducing ZMYND8 degradation.^92^ LINC00339 regulated TNBC cell growth by promoting proliferation and inhibiting apoptosis *via* regulation of miR‐377‐3p/HOXC6 expression.^93^ Moreover, there are also some non‐classical lncRNA‐involved mechanisms in the regulation of TNBC progression. MIR100HG was identified as a pro‐oncogene for TNBC progression with a high expression level in TNBC and reduced MIR100HG significantly inhibited cell proliferation and induced cell cycle arrest in the G1 phase. Furthermore, MIR100HG negatively regulated p27 gene expression to control the cell cycle by forming RNA‐DNA triplex structures, impacting the progression of TNBC.^94^ LncRNA NRAD1 was regulated by ALDH1A3 and was a therapeutic target for TNBC for its regulation of gene expression and effect on cancer cell survival.^95^ LncRNA DANCR enhanced PI3K/AKT signals and TNBC proliferation by binding to RXRA and increasing its serine 49/78 phosphorylation to activate PIK3CA transcription.^96^

In contrast, several lncRNAs also play tumour suppressor roles in TNBC progression. Downregulating lncRNA SONE resulted in a remarkable TP53 decrease and c‐MYC increase, which could alter the expression of downstream tumour suppressor miRNAs, leading to increased cell proliferation and migration.^97^ LncRNA RMST functioned as a tumour suppressor in TNBC by decreasing cell proliferation and migration, modulating the cell cycle and enhancing apoptosis.^98^ Moreover, lncRNA PTCSC3 inhibited TNBC cell proliferation by downregulating lncRNA H19, exhibiting a novel RNA‐RNA interacting mechanism in TNBC.^99^

#### 4.3.2 LncRNAs mediate migration and metastasis

As long non‐coding antisense transcript of NAMPT, NAMPT‐AS was upregulated in TNBC and promoted cell migration. NAMPT‐AS epigenetically regulated the expression of NAMPT in two different ways. One was that the transcription of NAMPT was activated by NAMPT‐AS, thereby recruiting POU2F2. The other was that NAMPT‐AS acted as ceRNA to rescue NAMPT degradation from miR‐548b‐3p.^10^ A previous report indicated that a novel miRNA sponge, lincRNA ROR, was dramatically upregulated in TNBC and interacted with miR‐145 to regulate cancer cell invasion by targeting ARF6.^11^ Wang et al^12^ found that linc‐ZNF469‐3 interacted with miR‐574‐5p and overexpression of linc‐ZNF469‐3 upregulated ZEB1 expression, which stimulated lung metastasis of TNBC. Furthermore, proteins of the MMP family have been shown to be involved in the breakdown of the extracellular matrix, which is essential for cancer metastasis. Shi et al^13^ demonstrated that lncRNA HULC expression was increased in TNBC tissues and silencing HULC expression effectively suppressed cell metastasis through suppressed MMP2 and MMP9 expression.

In addition, it has been proven that lncRNAs also function as cell migration inhibitors in various ways. LncRNA LOC554202 was downregulated in TNBC by promoter hypermethylation, and LOC554202 loss promoted cell migration, accounting for the aggressive phenotype of TNBC to some extent.^14^ In addition, lncRNA NEF was found to be downregulated in TNBC, and NEF overexpression inhibited cell migration by negatively regulating miR‐155.^15^

#### 4.3.3 LncRNAs dominate the sensitivity to chemotherapy

Studies have shown that lncRNA NEAT1 expression is upregulated in cisplatin‐ and taxol‐resistant cells compared with parental cells. By qRT‐PCR assay, it was demonstrated that when knocking down NEAT1 in sh‐NEAT1 cells, drug transporter genes, such as ATP7A and ATP7B, were downregulated, and functional analysis indicated that NEAT1 knockdown sensitized cells to chemotherapy through a synergistic effect.^16^ BORG, an oncogenic lncRNA, was greatly responsive to cytotoxic drug treatment, particularly doxorubicin. The mechanism of drug BORG‐associated resistance relied on its significant activation of the NF‐κB signalling pathway through the BORG‐mediated feed‐forward signalling loop and its ability to activate RPA1, making BORG‐expressing TNBC sensitive to doxorubicin‐induced cytotoxicity.^17^ In addition, upregulation of lncRNA HCP5 contributed to cisplatin resistance in TNBC, and inhibition of HCP5 reversed resistance to cisplatin by upregulating PTEN expression.^18^ However, tumour suppressive lncRNA GAS5 could enhance the sensitivity of TNBC to paclitaxel and induce apoptosis in TNBC cells by targeting miR‐378a‐5p/SUFU signalling.^19^

Moreover, lncRNAs not only participate in the process of chemoresistance, but can also be used for predicting the response to neoadjuvant chemotherapy in TNBC. Zheng et al^110^ systematically compared gene expression between TNBC patients with pathological complete response and those without a complete response to neoadjuvant chemotherapy and ultimately developed a gene signature of 2 coding genes and 3 lncRNAs to predict the response to neoadjuvant chemotherapy of TNBC patients.

#### 4.3.4 LncRNAs serve as potential biomarkers for diagnosis and prognosis

At present, with the deepening of TNBC research, lncRNAs have generally been revealed as promising diagnostic and prognostic biomarkers.^111^ ANRIL, HIF1A‐AS2 and UCA1 have been reported to be markedly upregulated in the plasma of patients with TNBC compared with patients with non‐TNBC. In addition, a 3‐lncRNA signature obtained using the logistic model exhibited excellent diagnostic values with an AUC of 0.934.^112^ In addition, an epigenome‐wide association study (EWAS) conducted by Bermejo et al^113^ revealed that hypermethylation of LINC00299 in the peripheral blood of TNBC patients could function as a useful circulating biomarker for TNBC and exhibited excellent diagnostic value. Jiang et al^114^ proposed an integrated mRNA‐lncRNA signature based on a combination of mRNA and lncRNA species and found that lncRNA HIF1A‐AS2 and AK124454 could be involved in mediating TNBC cell proliferation, invasion and paclitaxel resistance and also exhibited good prognostic value.

Furthermore, Lv et al^115^ compared the expression levels of lncRNAs in TNBC and non‐TNBC tissues separately and found that dysregulated lncRNAs participated in important biological processes. They further validated these lncRNA expression levels and confirmed that four dysregulated lncRNAs were significantly correlated with TNBC occurrence. Other systemic analyses revealed that seven prognosis‐related lncRNAs were significantly associated with poor RFS in TNBC patients.^116^

#### 4.3.5 LncRNAs mediate immunomodulation

Immunotherapy, including PD‐1 and/or PD‐L1 blockade, is an important cancer therapeutic method to restrict cancer progression.^117^ However, during immunotherapy, the loss of antigenicity and evasion of immune checkpoints in malignant tumour cells is a puzzling issue that deserves further exploration. Hu et al^118^ reported that tissue‐specific lncRNA LINK‐A expression facilitated the crosstalk between GPCR signalling and upregulated K48‐polyubiquitination‐mediated degradation of the antigen PLC and intrinsic tumour suppressors Rb and p53. Furthermore, the treatment with LINK‐A locked nucleic acids or GPCR antagonists stabilized the PLC components Rb and p53 and sensitized breast cancer cells to immune checkpoint blockers. In the clinical practice, TNBC patients with PD‐1 blockade resistance exhibited upregulated LINK‐A expression and downregulated PLC components. Although no additional literature has focused on this hotspot, lncRNA‐dependent immunomodulation is next promising research direction.

## 5 CIRCULAR RNAs

### 5.1 Biogenesis of circRNAs

**Figure 3:**
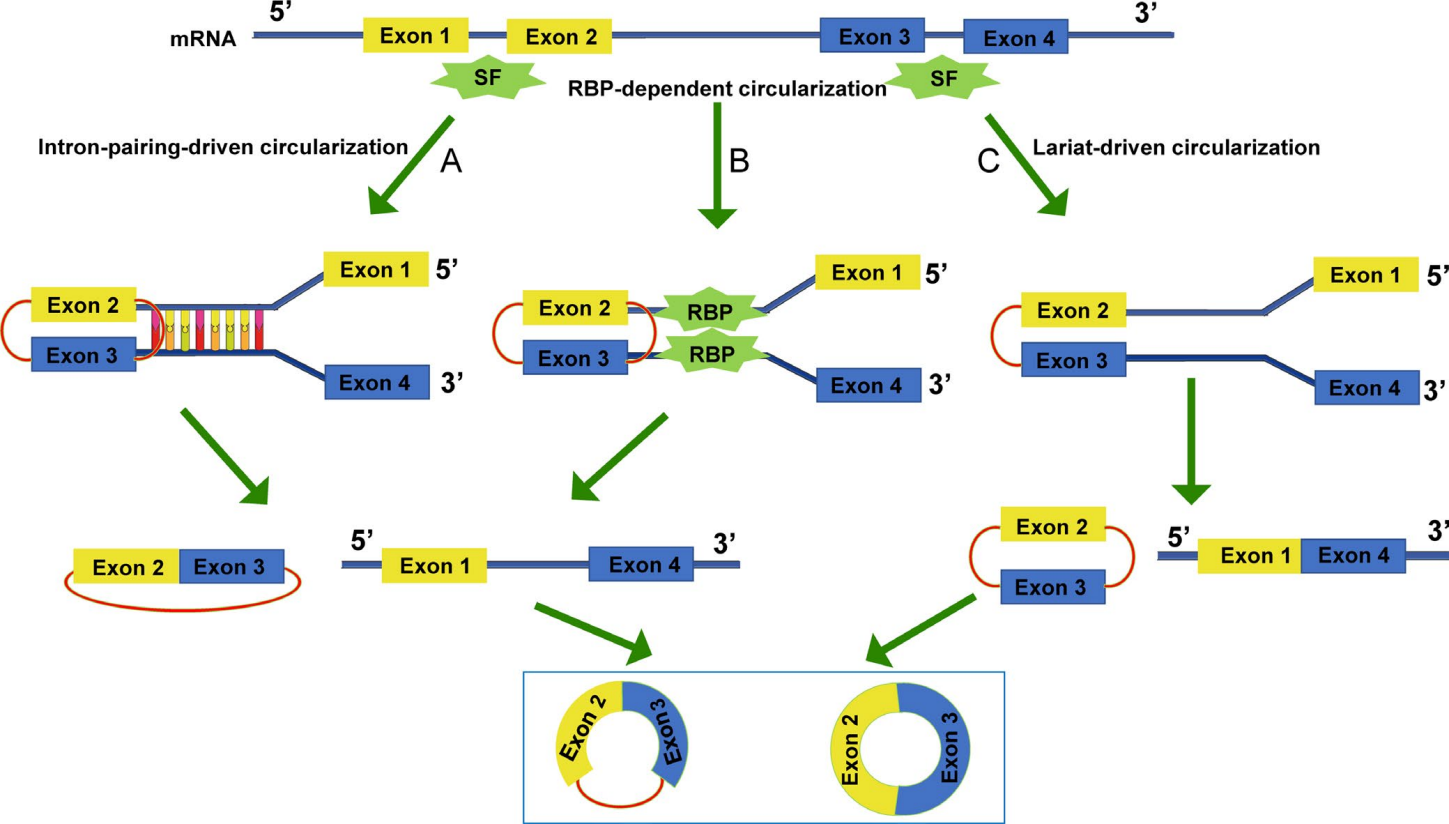
Mechanism of exonic circRNA and EIciRNA biogenesis. A, Intron‐pairing‐driven circularization: Complementary sequence motifs in introns flanking the exons can directly pair each other to induce circularization, and as a result, exonic circRNAs or EIciRNAs are produced; B, RBP‐dependent circularization: Interaction between RBPs bound to sequence motifs in both introns flanking the exons to be circularized facilitates the head‐to‐tail end‐joining of exon 2 and exon 3; C, Lariat‐driven circularization: Folding of a region of pre‐RNA can result in exon skipping; furthermore, the splice donor in 3' end of exon 1 and the splice acceptor in 5' end of exon 4 are covalently joined together to form a lariat containing exon 2 and exon 3, ultimately producing a circRNA. SF: splicing factor; RBP: RNA‐binding protein

**Figure 4:**
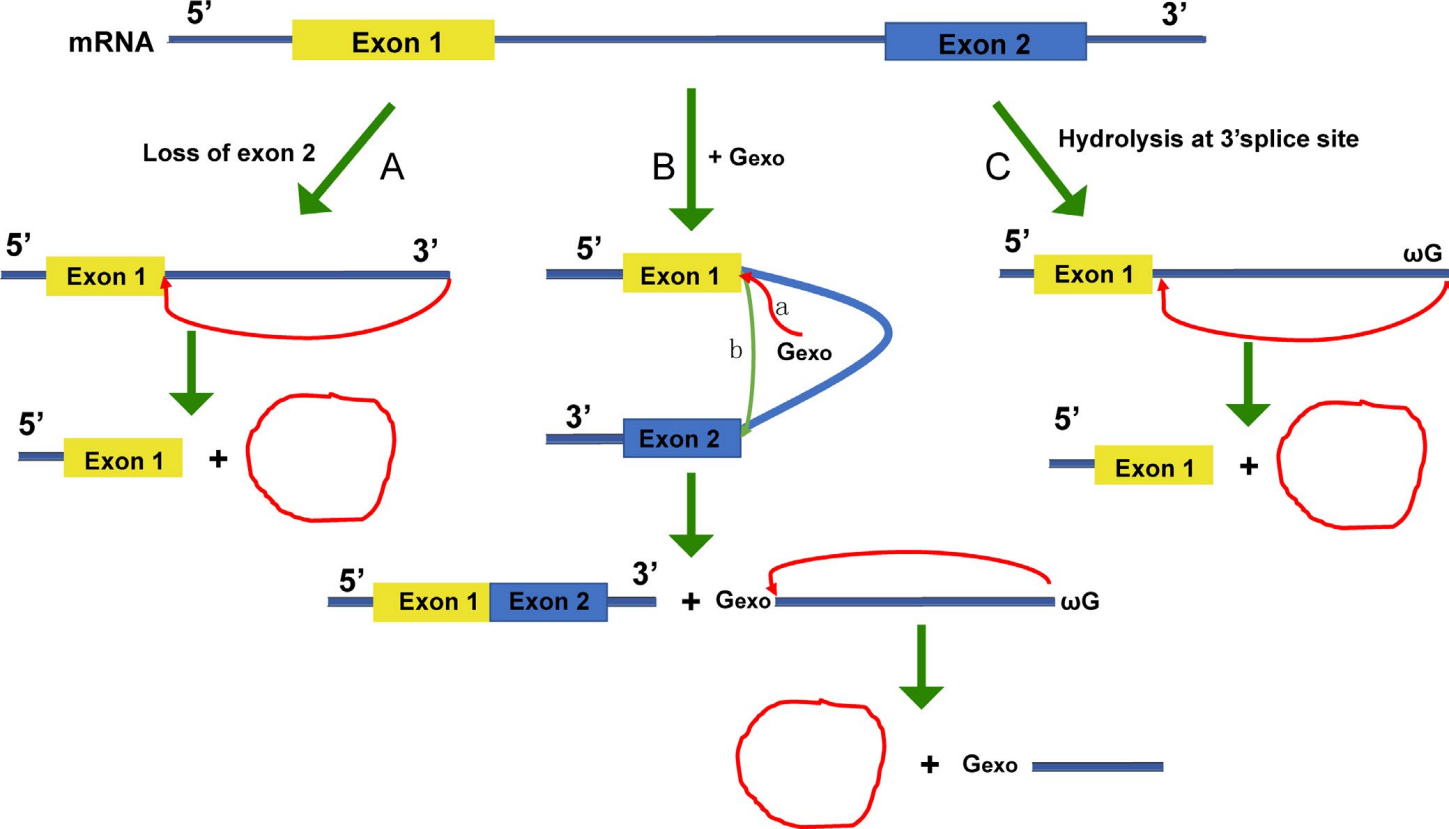
Mechanism of intronic circRNA biogenesis. A, Group II intron‐mediated circRNA formation: Circle formation requires prior to release of the 3’ end exon. The terminal 2’‐OH group of the intron attacks the 5’‐splice site, creating a circular RNA by 2',5'‐phosphodiester formation. B, Group I intron‐supported regular splicing: An exogenous guanosine (exoG) bound to the intron structure serves as a nucleophile attacking the 5’‐splice site. (a) Upon first transesterification, the 5’‐exon is cut‐off and exoG becomes linked to the intron. (b) The terminal 3’‐OH group of the 5’‐exon then attacks the 3’‐splice site, and the ligated exons and a linear intron are released. Eventually, the linear intron is circularized by nucleophilic attack of the 2’‐OH group of the terminal guanosine (ωG) onto a phosphodiester bond close to the 3′‐end and release of a short 3′‐tail. Note that in this case, a 2'‐5'‐phosphodiester bridge closes the circle. C, Circular intron RNA: Hydrolysis of the 3'‐exon allows circle formation by direct nucleophilic attack of ωG onto the 5’‐splice site, producing an RNA lariat circularized with a 2',5'‐phosphodiester

Circular RNA was first discovered in the 1970s; the first observation of circRNA in plant‐infected *Viroids* by electron microscopy was reported by Sanger et al in 1976.^119^ So far, scholars have found that more than 10% of protein‐encoding genes in a variety of biological cells and tissues can produce circRNAs,^120^ suggesting that circRNAs are ancient molecules that with evolutionary conservation. CircRNAs are derived from precursor mRNAs (pre‐mRNAs), which are transcribed by RNA polymerase II. CircRNAs can be divided into three subclasses according to their various position and forming mechanism, exonic circRNA, intronic circRNA and EIciRNA.^121^ Although increasing studies concentrated on the cycling processes, the exact mechanisms of circRNAs maturation have not been fully elucidated. At present, three hypothetical models explain the formation of exonic circRNA and/or EIciRNA: intron‐pairing‐driven circularization, RNA‐binding protein (RBP)‐dependent circularization and lariat‐driven circularization (Figure 3).^122^ There are also three hypothetical models expounding the formation of intronic circRNA have been issued: Group II intron‐mediated circRNA formation, group I intron‐supported regular splicing and circular intron RNA (ciRNA; Figure 4).^123^, ^124^

### 5.2 Functions of circRNAs

#### 5.2.1 CircRNAs act as ceRNA or miRNA sponges

Similar to lncRNA, the most common mechanism of circRNA regulating biological process is the ceRNA model. An increasing number of studies have proven that multiple circRNAs act as miRNA sponges. For example, circTP63 functioned as a ceRNA to upregulate FOXM1, thus promoting lung cancer progression.^125^ CircHIPK3 mediated autophagy *via* sponging miR‐124‐3p to regulate STAT3/PRKAA/AMPKα signalling in STK11‐mutant lung cancer.^126^ CircMYLK promoted hepatocellular carcinoma progression by upregulating Rab23 via sponging miR‐362‐3p.^127^ To sum up, these studies above support the idea that circRNAs functioning as miRNA sponges may be a common mechanism in cancerous diseases.

#### 5.2.2 CircRNAs regulate gene transcription

Although most research focuses on the role of circRNAs as sponges for miRNAs, some scholars have uncovered that some intronic circRNAs and EIciRNAs could regulate protein level by regulating gene expression at transcriptional or post‐transcriptional level. Logically, these circRNAs always exist in the nucleus. For example, Li et al^128^ found that circEIF3J and circPAIP2 upregulated the expression of their parental genes in *cis* and raised a novel regulatory strategy for transcriptional control *via* specific RNA‐RNA interaction between U1 snRNP and EIciRNAs. CiRNAs can also regulate gene transcription. Besides, intronic circRNAs also participate in gene regulation. Scholars uncovered that ci‐ANKRD52 and ci‐SIRT7 can act as positive regulators to upregulate their parental gene transcription *via* interacting with RNA polymerase II.^123^

#### 5.2.3 CircRNAs interact with functional proteins

Similar to linear RNAs, which have been reported to interact with proteins, several studies have also exhibited that some circRNAs can work as protein partner such as circANRIL and circFOXO3. CircANRIL had been revealed to directly bind to PES1, an important 60S‐preribosomal assembly factor and next control maturation of ribosomal RNA and modulating pathways of atherogenesis, resulting in regulating the progression of atherosclerosis.^129^ Besides, circFOXO3 was found to be highly expressed in non‐cancer cells and correlated with cell cycle progression. Functional analysis indicated that overexpression of circFOXO3 can repress cell cycle progression *via* binding to CDK2 and cyclin‐dependent kinase inhibitor 1 (or p21), resulting in the formation of a ternary complex.^130^

#### 5.2.4 CircRNAs can be translated into proteins

Most circRNAs are derived from exons and predominantly present in the cytoplasm, suggesting that they can be loaded into ribosomes for translation into polypeptides. CircZNF609 was derived from the circularization of the second exon of its host gene. A 753 nt ORF was present in circZNF609, which could be translated into a protein in a splicing‐dependent and cap‐independent manner, providing an novel example of a protein‐coding circRNA in eukaryotes.^131^ Ivano et al^132^ revealed that circFBXW7 was highly expressed in the normal human brain tissues, which can be translated into novel 21 kDa protein termed as FBXW7‐185aa. Besides, upregulation of FBXW7‐185aa inhibited proliferation and cell cycle acceleration in tumour cells. In addition, circSHPRH can be translated into 17 kDa named SHPRH‐146aa. Both circSHPRH and SHPRH‐146aa were abundantly expressed in normal human brains and downregulated in glioblastoma tissues. The re‐expression of SHPRH‐146aa in glioblastoma cells suppressed their malignant behaviour and tumorigenicity in vitro and in vivo.^133^ As can be seen from the above examples, the definition of circRNA as ncRNA is somewhat limited.

### 5.3 CircRNAs in TNBC

In recent years, circRNAs have gradually become a novel hotspot in the ncRNAs and cancer research field. However, the functions of circRNAs in cancerous diseases, especially in TNBC, have not been fully understood. The known studies about circRNAs mostly focalized on the mechanisms of ceRNA in TNBC progression. Besides, circRNAs act as biomarkers for diagnosis and prognosis in TNBC is also widely observed (Table 4).

**Table 4:** Summarization of the cellular functions of circRNAs in tumorigenesis of TNBC

CircRNAs	Circbase ID	Role in TNBC	Cancer phenotype	Sponge miRNAs	Target genes	References
circGFRA1	hsa_circ_005239	Oncogene	Promoted proliferation and induced apoptosis	miR‐34a	GFRA1	^134^
circEPSTI1	hsa_circ_000479	Oncogene	Promoted proliferation and induced apoptosis	miR‐4753 miR‐6809	BCL11A	^135^
circUBAP2	hsa_circ_0001846	Oncogene	Promoted proliferation and migration and induced apoptosis	miR‐661	MTA1	^136^
circAGFG1	hsa_circ_0058514	Oncogene	Promoted proliferation, migration and invasion	miR‐195‐5p	CCNE1	^137^
circKIF4A	hsa_circ_0007255	Oncogene	Promoted proliferation and migration	miR‐375	KIF4A	^138^
circPLK1	hsa_circ_0038632	Oncogene	Promoted proliferation and migration	miR‐296‐5p	PLK1	^139^
circRAD18	hsa_circ_0002453	Oncogene	Promoted proliferation and migration and induced apoptosis	miR‐208a/miR‐3164	IGF1/FGF2	^140^
circRNA_069718	has_circ_069718	Oncogene	Promoted proliferation and invasion	/	/	^141^
circANKS1B	hsa_circ_0007294	Oncogene	Promoted migration and invasion	miR‐148a‐3p miR‐152‐3p	USF1	^142^
ciRS‐7	/	Oncogene	Promoted migration and invasion	miR‐1299	MMPs	^143^
circITCH	/	Tumour suppressor	Inhibited proliferation, migration and invasion	miR‐214/miR‐17	ITCH1	^144^
circTADA2A‐E6	hsa_circ_0006220	Tumour suppressor	Inhibited proliferation, migration and invasion	miR‐203a‐3p	SOCS3	^145^
circFBXW7	hsa_circ_0001451	Tumour suppressor	Inhibited proliferation, migration and invasion	miR‐197‐3p	FBXW7	^146^

#### 5.3.1 CircRNAs act as tumour promoters

Derived from gene GFRA1, circGFRA1 was upregulated in TNBC and high expression of circGFRA1 was correlated with poor OS. Knockdown of circGFRA1 suppressed proliferation and promoted apoptosis via binding to miR‐34a and upregulating GFRA1 expression in TNBC.^134^ Besides, silencing of circEPSTI1 inhibited cell proliferation and induced apoptosis via sponging miR‐4753 and miR‐6809 to increase BCL11A in TNBC.^135^ It was also revealed that circUBAP2 was correlated with tumour size, advanced TNM stage and worse prognosis in TNBC and promoted tumour progression by sponging miR‐661 to upregulate MTA1.^136^ Furthermore, circAGFG1, circKIF4A, circPLK1 and circRAD18 were revealed to promote malignant progression of TNBC by sponging corresponding tumour suppressive miRNAs as well.^137^, ^138^, ^139^, ^140^ In addition, Zhang et al^141^ found that circRNA_069718 can promote the proliferation and invasion of cancer cells by activating the Wnt/β‐catenin pathway, thereby promoting the malignant progression of TNBC.

On the other hand, there is emerging evidence that circRNAs are associated with the metastasis of TNBC. Zeng et al^142^ found that circANKS1B was remarkably upregulated in TNBC tissues and increased circANKS1B expression was correlated with lymph node metastasis. Functional studies uncovered that circANKS1B promoted breast cancer cell migration both in vitro and in vivo, whereas it had no effect on breast cancer growth *via* sponging miR‐148a‐3p and miR‐152‐3p to upregulate USF1, leading to transcriptional activation of TGF‐β1, which could upregulate TGF‐β1/Smad signalling to promote EMT. In addition, Sang et al^143^ found that circRNA ciRS‐7 was highly expressed in TNBC tissues and upregulated the expression of multiple MMPs by sponging miR‐1299, promoting tumour invasion and metastasis in vitro and in vivo.

#### 5.3.2 CircRNAs act as tumour suppressors

Several studies have found that circRNAs function as tumour suppressors in TNBC as well. CircITCH was remarkably downregulated in TNBC tissues and predicted poor prognosis. Overexpression of circITCH significantly suppressed cell proliferation and migration *via* acting as a sponge for miR‐214 and miR‐17 to upregulate ITCH1, thus inactivating Wnt/β‐catenin signalling.^144^ Based on screening circRNA profiles, Xu et al^145^ identified circTADA2A‐E6 spliced from exon 6 of TADA2A gene as a prognostic biomarker in TNBC and overexpression of circTADA2A‐E6 significantly inhibited cell proliferation, migration and clonogenicity *via* regulating miR‐203a‐3p/SOCS3 axis. Interestingly, circRNA‐encoded proteins were also involved in TNBC progression. CircFBXW7 inhibited the malignant progression of TNBC by sponging miR‐197‐3p and encoding a tumour suppressor FBXW7‐185aa.^146^ To sum up, limited numbers of studies on the tumour suppressor roles of circRNAs in TNBC are available currently, so it is still urgent to explore further mechanism of circRNAs participating in the suppression of TNBC.

## 6 OTHER NON‐CODING RNAs

Although most research related to TNBC are concentrated on the field of miRNAs, lncRNAs and circRNAs, other ncRNAs also have great significant functions in the carcinogenesis of TNBC. TDR is one of the other concentrated ncRNAs in TNBC. TRNA has been for a long time deemed to transcripts with non‐coding capacity, but with well‐established functions in the translation process. However, due to the discovery of tRNA fragments with the function that can regulate gene expression, their crystallized roles have changed over the last decade. Recently, tDRs have been detected in several human diseases and biological processes, including TNBC.^147^, ^148^ TDR‐000620 was found to downregulated in TNBC stem cells by RNA sequencing and validation of qPCR. Besides, low tDR‐000620 expression served as an independent predictive factor for RFS of TNBC patients.^149^ Besides, the expression of tDRs from specific tRNA loci has been found to be associated with the observed race disparities in TNBC, such as the nuclear tRNA^Gly^ and tRNA^Leu^, the mitochondrial tRNA^Val^ and tRNA^Pro^.^150^ In addition to serving as biomarkers, tDRs also participate in the process of TNBC. Cui et al^151^ found that tDR‐0009 and tDR‐7336 were notably upregulated in the SUM‐1315 cell lines stimulated by hypoxia; further, bioinformatic analysis indicated that these two upregulated tDRs might be involved in the chemoresistance to doxorubicin in TNBC via mediating the activation of phosphorylation of STAT3. As for other ncRNAs, such as snoRNA and snRNA, their tumour‐associated functions have been observed in multiple cancers,^152^, ^153^, ^154^ but no exact roles have been defined in TNBC, which need to be further explored in the future.

## 7 PERSPECTIVES IN CLINICAL PRACTICE

### 7.1 NcRNAs as diagnostic and prognostic biomarkers in cancer assessment

Non‐coding RNAs were demonstrated to have strong diagnostic and prognostic values in multiple cancers, which were revealed by pan‐cancer analysis.^155^, ^156^ With the development of RNA‐sequencing technology and advanced analysis methods, the roles of ncRNAs have been well summarized. Guo et al^157^ performed small RNA profiling of 26 TNBC cell lines and compared the abundance of ncRNAs among the transcriptional subtypes of TNBC, which identified a mass of dysregulated small ncRNAs, highlighting potential biomarkers for future studies. Although there are still quite a few challenges that need to be addressed, the roles of ncRNAs in clinical practice are being a novel hotspot and many scholars try to employ ncRNAs as biomarkers and therapeutic targets to diagnose, treat and monitor TNBC. As mentioned before, lots of ncRNAs are dysregulated in TNBC tissues compared with non‐TNBC and healthy samples, which might serve as potential biomarkers to diagnose TNBC. However, more value will be aggrandized if dysregulated ncRNAs can detected in the peripheral blood. Circulating miR‐199a‐5p, miR‐105, miR‐93‐3p and miR‐200c had been reported to serve as TNBC‐specific diagnostic biomarkers.^21^, ^22^, ^23^ Besides, the dysregulated lncRNA ANRIL, HIF1A‐AS2 and UCA1 can be detected in plasma of TNBC patients and the 3‐lncRNAs combined signature exhibited excellent diagnostic value.^112^ Moreover, even hypermethylation of LINC00299 in peripheral blood of TNBC patients also served as a useful circulating biomarker for TNBC.^113^ However, the circulating circRNAs have not been found to be biomarkers in TNBC. Theoretically, circRNAs are covalently closed and generally resistant to the degradation of ribonucleases, suggesting they might be more stabilized in peripheral blood. Thus, detection of the dysregulated circRNAs in the peripheral blood of TNBC patients should next hotspot in the field of ncRNA research.

### 7.2 NcRNAs as therapeutic tool in cancer treatment

It has been proven that abnormal small ncRNA expression levels in tumour cells can affect tumour development and therefore can be used as effective targeted drugs for the treatment of tumours.^158^ Although small ncRNAs have been shown to be promising and effective therapeutic drugs in vitro, due to the degradation of nucleases in the body, naked ncRNAs have a short half‐life in the blood, and the low bioavailability of these nucleic acid drugs in vivo is a major challenge. Therefore, ncRNAs need to be transported to the target tissue by a suitable carrier to exert their effect. Various small ncRNA carriers or systems have been proposed and widely explored, including nanoparticles, ncRNA modification and oncolytic adenovirus strategies.^159^ Nanoparticle‐based small ncRNA carriers are the most common strategies. Shu et al^160^ developed a 15 nm nanoparticle with a 58 nt phi29 pRNA‐three‐way junction (3WJ), an 8 nt sequence complementary to the seed region of miR‐21 and a 39 nt EGFR targeting aptamer. They successfully applied this novel RNA nanotechnology for efficient delivery of anti‐miR‐21 to block the growth of TNBC in orthotopic mouse models. Moreover, Yin et al^161^ utilized the thermodynamically and chemically stable 3WJ motif as a scaffold to carry an RNA aptamer binding to CD133 and a locked nucleic acid sequence for miR‐21 inhibition to develop a unique delivery strategy to control TNBC progression. Although ncRNA modification and oncolytic adenovirus strategies are also effective technology to enhance the precision and durability of small ncRNAs in targeting functional genes in cancer, the relevant research on their utility in TNBC is not currently available. Thus, continued progress in the development of ncRNA carrier strategies might allow these approaches to be important and powerful alternative tools to treat TNBC.

## 8 CONCLUSION

**Figure 5:**
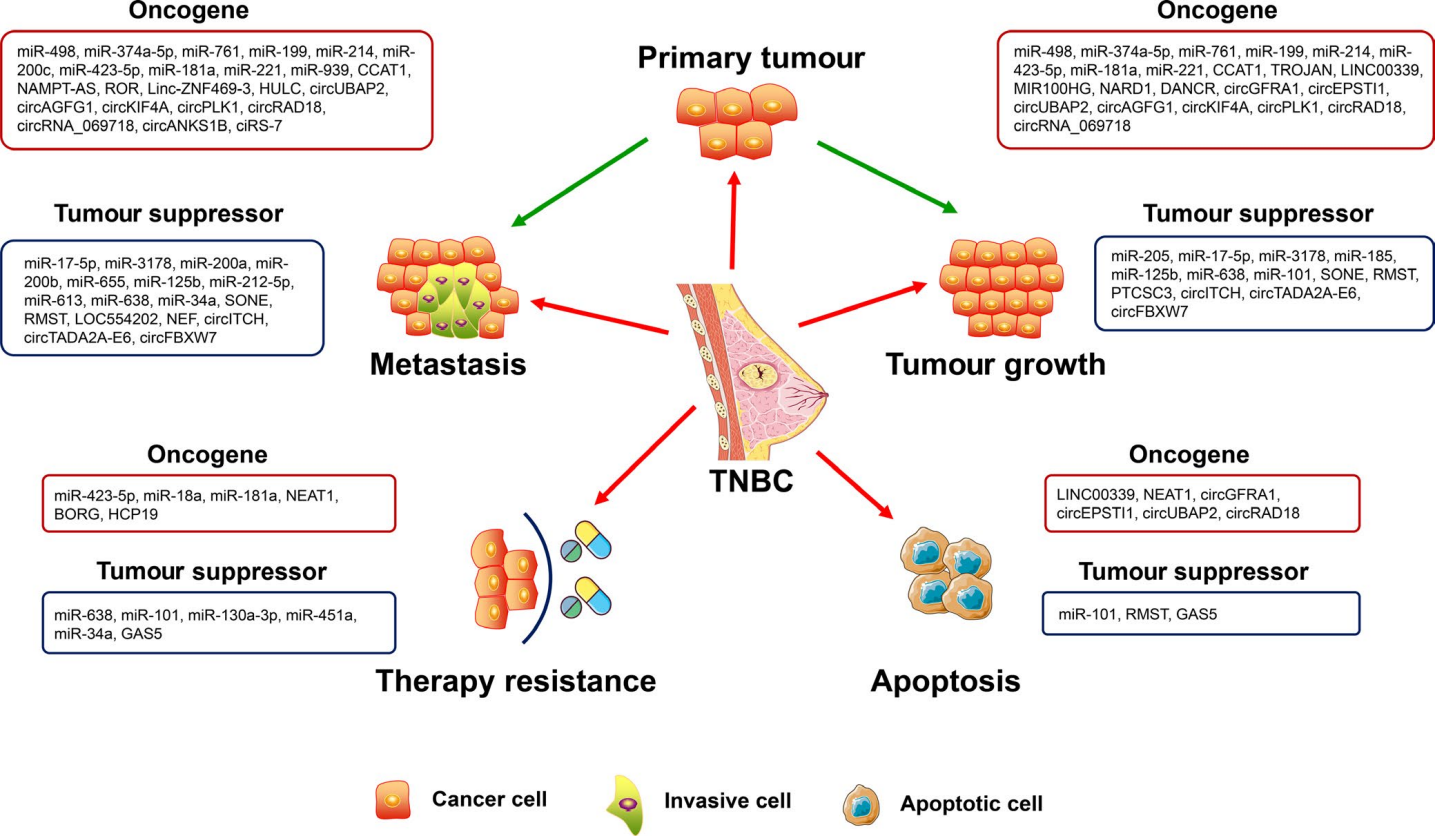
Summary of the function of ncRNAs in TNBC. NcRNAs play multifaceted roles in tumour initiation and development, which can control cell proliferation, apoptosis, migration and therapy resistance by orchestrating their downstream targets. Obviously, one ncRNA tends to affect tumour progression by regulating multiple attributes of TNBC cells

To conclude, ncRNAs have notable effects on TNBC progression. We summarize the roles of ncRNAs and their mechanisms in TNBC in Figure 5. Although the functions of miRNAs and lncRNAs on the carcinogenesis and development of TNBC have been widely studied and have well‐established roles, many other ncRNAs including circRNAs also have a significant influence on tumour progression but have long been neglected. With more research interests concentrated on the lesser‐known ncRNAs and their relationship with TNBC, we believe that the mystery of the ncRNA world will eventually be solved and the clinical practice of TNBC management will be largely improved in the future.

## CONFLICT OF INTEREST

The authors declare that they have no competing interests.

## AUTHOR CONTRIBUTIONS

YZ, CL and JM conceptualized the review. JM and LH wrote the manuscript. LH, HW, RX and YL prepared the figures and tables. YZ and CL critically reviewed and edited the manuscript. All authors read and approved the final manuscript.

## Data Availability

The data that support the findings of this study are available from the corresponding author upon reasonable request.
